# Genetics and functions of the retinoic acid pathway, with special emphasis on the eye

**DOI:** 10.1186/s40246-019-0248-9

**Published:** 2019-12-03

**Authors:** Brian Thompson, Nicholas Katsanis, Nicholas Apostolopoulos, David C. Thompson, Daniel W. Nebert, Vasilis Vasiliou

**Affiliations:** 1 Department of Environmental Health Sciences, Yale School of Public Health, 60 College St, New Haven, CT 06520 USA; 2 Stanley Manne Research Institute, Lurie Children’s Hospital, Chicago, IL 60611 USA; 3 Departments of Pediatrics, Northwestern University Feinberg School of Medicine, Chicago, IL 60611 USA; 4 Department of Clinical Pharmacy, Skaggs School of Pharmacy and Pharmaceutical Sciences, University of Colorado Denver, Aurora, CO 80045 USA; 5 Department of Environmental Health and Center for Environmental Genetics, University Cincinnati Medical Center, Cincinnati, OH 45267-0056 USA

**Keywords:** Retinoic Acid, Eye Development, gnomAD, ExAC

## Abstract

Retinoic acid (RA) is a potent morphogen required for embryonic development. RA is formed in a multistep process from vitamin A (retinol); RA acts in a paracrine fashion to shape the developing eye and is essential for normal optic vesicle and anterior segment formation. Perturbation in RA-signaling can result in severe ocular developmental diseases—including microphthalmia, anophthalmia, and coloboma. RA-signaling is also essential for embryonic development and life, as indicated by the significant consequences of mutations in genes involved in RA-signaling. The requirement of RA-signaling for normal development is further supported by the manifestation of severe pathologies in animal models of RA deficiency—such as ventral lens rotation, failure of optic cup formation, and embryonic and postnatal lethality. In this review, we summarize RA-signaling, recent advances in our understanding of this pathway in eye development, and the requirement of RA-signaling for embryonic development (e.g., organogenesis and limb bud development) and life.

## Background

For human health, the importance of retinol, also known as vitamin A, has been known since ancient times, when the practice of squeezing liver juice into the eye was used as a treatment for night blindness [1]. The link between night blindness (nyctalopia) and nutrition was first described by Hippocrates during the 4th century BC, when he recommended eating raw liver in combination with honey as a cure [2].

The idea that certain foods possessed curative properties was understood for much of human history. However, it was not until much later that a series of controlled experiments (i.e., human dietary supplementation and animal models during the early twentieth century) allowed scientists to investigate how removal of certain factors from the diet could cause debilitating illnesses and death [3–5]. Biochemical experiments in vertebrate models subsequently revealed that retinol was the active compound involved in cell growth and development, along with its precursors and metabolites [6].

The aldehyde derivative of retinol, 11-*cis*-retinal, is required for vision [*reviewed in* [7]]. All-*trans*-retinoic acid (ATRA), the acid derivative of retinol, is able to prevent developmental defects in vitamin A-deficient animals [8]. The demonstration that retinoic acid (RA) could not be converted back to retinol in vivo led to the conclusion that RA was a necessary nutrient involved in cell growth and development [9]. Ultimately, a set of compounds and their metabolites with biological functions similar to retinol were termed “retinoids” [10].

Although the chemical structures of retinoids were identified in the early 1900s, little was known about the mechanisms by which these small lipophilic molecules exerted their biological effects [6]. A short time later, studies performed in vitamin A-deficient rats revealed that retinol supplementation stimulated RNA synthesis in intestinal cells [11]. Biochemical studies subsequently identified serum, membrane, and cytosolic proteins that were essential for retinol transport, uptake, and metabolism [12]. Examples include retinol-binding protein 4 (RBP4), stimulated-by-retinoic-acid 6 (STRA6) membrane receptor, and the cellular retinol-binding protein (CRBP) family, also known as RBP1 and RBP2 [12].

Experiments carried out in chick and mouse embryos identified RA as the active metabolite of vitamin A that possessed the ability to regulate cellular differentiation and proliferation, as well as pattern formation during embryogenesis [*reviewed in* [13]]. At the molecular level, the action of RA is mediated by two distinct classes of proteins: (i) a family of nuclear receptors comprising RA receptors (RARs) and retinoid X receptors (RXRs) that regulate gene transcription in a ligand-dependent fashion and (ii) a family of cytosolic proteins called cellular retinoic acid-binding proteins (CRABP1 and CRABP2) which facilitate cellular RA uptake and nuclear transfer [14]. These studies provided a link between the chemical structure of retinoids and their biological action.

Although the developmental role of RA has been extensively studied in model organisms [*reviewed in* [13]], little is known about the exact role of RA in human development. Currently, almost all our molecular understanding about the pathogenesis of RA deficiency is based on either vertebrate animal knockout models (in which genes encoding proteins involved in RA synthesis or degradation are selectively inactivated) or experiments involving rodents fed diets deficient in vitamin A*.* Such studies have shown RA to be involved in early axial and central nervous system patterning, neurogenesis, regulation of limb bud development, and organogenesis [[3] and *reviewed in* [13, 14]]. To date, none of these studies has been systematically validated in humans due to ethical concerns and the difficulty of performing such clinical experiments. Nevertheless, based on observational studies in humans consuming vitamin A-deficient diets, it is well established that vitamin A is required in humans (even into adulthood) because it regulates fertility, maintains normal vision, inhibits neoplastic growth, and prevents neurodegenerative diseases [15].

With the recent publication of whole-exome sequencing (WES) data from ≈ 140,000 individuals by the Genome Aggregation Database (gnomAD) [16], it is now possible to investigate genetic intolerance to protein-truncated variants (PTVs) in a large population, i.e., to detect genes that are essential for human development. In this review, we first provide an overview of canonical and non-canonical RA metabolism (i.e., synthesis and catabolism) and the mechanism of RA target gene regulation. We then provide an update on the role of RA-signaling in eye development in mouse and zebrafish and discuss the ocular diseases in humans who have mutations in genes involved in the RA-signaling pathway—such as microphthalmia, coloboma, and anophthalmia. Finally, we take advantage of population-level variation databases to identify which genes involved in the RA pathway display loss-of-function intolerance, thus indicating their requirement for human development and life.

## Main text

### Retinoic acid synthesis, catabolism, and gene regulation

**Fig. 1:**
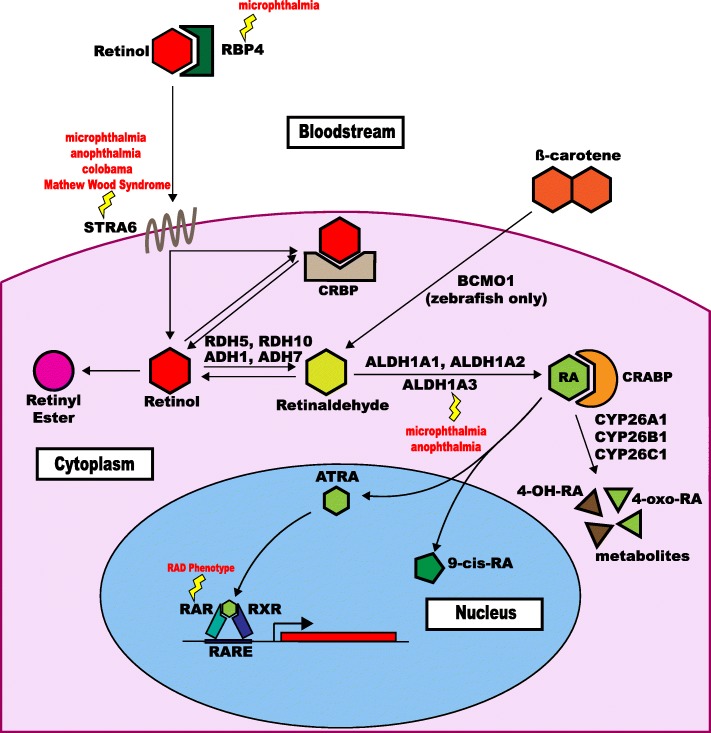
Retinoic acid (RA)-signaling pathway. Extracellular binding and import of retinol, retinol conversion, oxidation of retinaldehyde to ATRA and/or 9-cis-RA, binding to RA receptors (RAR, RXR), degradation of RA to metabolites, and activation/repression of retinoic acid response elements (RARE). This diagram shows the activation (Arrow) of a target gene (Red box). Lightning bolts indicate genes with associated human eye development diseases. Abbreviations: RBP4, retinol-binding protein 4; STRA6, stimulated by retinoic acid 6; RDH5, retinol dehydrogenase 5; RDH10, retinol dehydrogenase 10; ADH1, alcohol dehydrogenase 1; ADH7, alcohol dehydrogenase 7; CRBP, cellular retinol-binding protein; ALDH1A1, aldehyde dehydrogenase 1 family member A1; ALDH1A2, aldehyde dehydrogenase 1 family member A2; ALDH1A3, aldehyde dehydrogenase 1 family member A3; CYP26A1, cytochrome P450 family 26 subfamily A member 1; CYP26B1, cytochrome P450 family 26 subfamily B member 1; CYP26C1, cytochrome P450 family 26 subfamily C member 1; ATRA, all-*trans*-retinoic acid ; 9-cis-RA; RAR, retinoic acid receptor ; RXR, Retinoic X Receptor; RARE, retinoic acid response elements.

This section describes the components of the retinoic acid signaling pathway including cellular uptake of retinol, conversion of retinal to retinaldehyde, retinaldehyde oxidation to RA, RA degradation, and target gene activation (Fig. 1).

#### Canonical pathway of RA synthesis

Early studies revealed that retinoids could not be synthesized de novo in most animals [*reviewed in* [13]]. Hence, the major source of retinoids during embryonic and fetal development is through placental transfer of maternal retinol. Postnatally, retinoids are primarily derived from the dietary intake of (i) carotenoids (such as β-carotene) contained in plant pigments and (ii) retinyl esters from animal sources, such as fish-liver oils, eggs, milk, and butter. Following ingestion, retinyl esters are hydrolyzed to retinol by intestinal mucosal enzymes, whereas carotenoids are cleaved into retinal and subsequently reduced to retinol or oxidized to RA. Retinol homeostasis is tightly regulated. As such, much of the synthesized retinol is converted back into retinyl esters for storage in liver hepatocytes and stellate cells. When needed, these esters are cleaved and released into the bloodstream as retinol [17, 18]. Upon release into the bloodstream, retinol is bound by retinol-binding protein 4 (RBP4).

Cells can take up the retinol-RBP4 complex *via* transmembrane receptor protein stimulated by retinoic acid 6 (STRA6), the product of the RA-inducible mouse gene *Stra6* (or human *STRA6* gene) [19]. The complex tissue-specific expression pattern of this gene during development influences which tissues are able to take up retinol [20]. Once inside the cell, two sequential reactions are required to transform retinol into retinaldehyde and RA (Fig. 1).

The first reaction is mediated by two classes of enzymes: (i) cytosolic alcohol dehydrogenases (ADHs) that belong to the medium-chain dehydrogenase/reductase family and/or (ii) microsomal retinol dehydrogenases (RDHs) that belong to the short-chain dehydrogenase/reductase family [21]. Initial studies in mice indicated that this reaction was catalyzed by ADH7 in the embryo [22, 23]; however, tissue-specific RDH10 is now believed to play the most important role in RA synthesis during development because mice expressing mutant *Rdh10* (RDH10^trex^) (that lacks the ability to convert retinol to retinal) display embryonic lethality [24]. Some degree of RA activity persists in mice expressing RDH10^trex^ (revealed by limited RARE-lacZ reporter activity at E9.5), indicating that other enzymes (such as ADH7) are able to generate retinaldehyde, albeit at lower levels (that do not support embryonic development) [24]. In addition, transgenic suppression of ADH5 (an enzyme ubiquitously expressed in embryo and adult) or of tissue-specific ADH1 and ADH7 revealed that ADH enzymes may have a role in controlling removal of excess retinol, rather than participating in RA synthesis per se [25].

The second reaction is oxidation of retinaldehyde to RA. This is catalyzed by three aldehyde dehydrogenases: ALDH1A1, ALDH1A2, and ALDH1A3 which are encoded (respectively) by *Aldh1a1*, *Aldh1a2*, and *Aldh1a3* (respectively) in mice, or by *ALDH1A1*, *ALDH1A2*, and *ALDH1A3* in humans. Each ALDH displays a distinct expression pattern that closely correlates with RA activity and with the dynamics of RA-signaling. ALDH1A2 is responsible in the mouse for almost all RA production during early embryogenesis, i.e., until ~E8.5 [13]. During gastrulation, ALDH1A2 is expressed mainly along the primitive streak and in mesodermal cells in the posterior end of the embryo [26]. Later, ALDH1A2 is expressed in the somatic and lateral mesoderm, posterior heart tube, and rostral forebrain—and subsequently in prospective cervical and trunk levels during body axis extension [26]. Thereafter, ALDH1A3 is responsible for RA synthesis in the eye and olfactory system. ALDH1A1, thought to be partly redundant with ALDH1A3, has been demonstrated to act only during eye development [27, 28]

#### Alternative pathway of RA synthesis

An alternative pathway for RA synthesis (elucidated in zebrafish) involves conversion of β-carotene to retinaldehyde by a β-carotene cleaving enzyme, β-carotene 15,15'-oxygenase 1 (BCO1) [29]. This pathway, believed to be an ancestral pathway from early chordates, is found mainly in marine fish in which retinaldehyde and carotenoids stored in the egg yolk are the main source of retinoids during development [29]. The mouse and human homolog, β-carotene 15,15'-oxygenase 1 (encoded by *Bco1; BCO1*, previously known as *BCDO1*), is expressed in retinal pigment epithelium (RPE)—as well as in kidney, intestine, liver, brain, stomach, and testis [30–32]. Its primary function is to generate retinaldehyde in photoreceptor cells and to supplement retinoid pools in other tissues [33].

In addition, a second β-carotene cleavage enzyme expressed in rabbits, β-carotene 9',10'-dioxygenase (encoded by the *BCO2* gene), catalyzes the cleavage of β-carotene into β-apocarotenoic acid, which can be transformed into RA without any involvement of ALDHs [34]. Lastly, cell culture studies have revealed that CYP1B1, a member of the cytochrome P450 family, can catalyze conversion of retinol to retinaldehyde and RA. It remains to be seen if this enzyme meaningfully contributes to RA synthesis in mammals [35].

#### RA catabolism

Cellular levels of RA must be tightly regulated to prevent RA toxicity [*reviewed in* [36]]; this can occur through control of RA synthesis and RA catabolism (Fig. 1). RA is converted into polar derivatives (4-hydroxy-RA and 4-oxo-RA) by the cytochrome P450 26 subfamily of enzymes, specifically CYP26A1, CYP26B1, and CYP26C1 [37–39]. Lethality occurs in CYP26A1, CYP26B1, and CYP26C1 null mouse models [40].

Although it was originally shown that the RA CYP26-mediated polar derivative—4-oxo-RA—can interfere with embryonic development when delivered exogenously by binding to and activating RARs [41], more recent in vivo data suggest that CYP26-mediated catabolism is required for embryonic development because its removal of RA prevents inappropriate signaling in specific tissues [42]. The CYP26 enzymes display an expression pattern which matches that of the ALDHs during embryogenesis; their targeted disruption causes teratogenic effects similar to those seen in RA toxicity. In mice, *Cyp26a1* and *Cyp26c1* are the first genes to be expressed in the rostral-most embryonic epiblast, whereas *Cyp26b1* is expressed in tail bud tissues and in the distal limb bud mesenchyme. Later in development, these enzymes display differential expression patterns in various developing organs, such as retina, dental epithelium, and inner ear [*reviewed in* [25]].

#### RA gene activation or repression

RA acts as an agonist of two nuclear receptor families that bind DNA and directly regulate transcription (Fig. 1). These families are (i) the RA receptors, i.e., retinoic acid receptor alpha (RARA), retinoic acid receptor beta (RARB) and retinoic acid receptor gamma (RARG), and (ii) the retinoid X receptors, i.e., retinoid X receptor alpha (RXRA), retinoid X receptor beta (RXRB), and retinoid X receptor gamma (RXRG) [*reviewed in* [43]]. The RARs are highly conserved in vertebrates and are primarily activated by all-*trans*-RA (ATRA). By contrast, the RXRs are activated by 9-*cis*-RA, a stereoisomer of ATRA that is detected only when vitamin A is in excess. RXRs are thought to act as heterodimeric scaffolding proteins that facilitate binding of the RAR-RXR complex to DNA—complex demonstrates greater affinity for DNA than the RAR or RXR homodimers [44–46]. RARA, RXRA, and RXRB are widely expressed in tissues, suggesting that most tissues are potential targets of RA [*reviewed by* [47]]. Mouse knockout studies involving the RAR and RXR families have shown developmental abnormalities when two or more receptors are inactivated with the exception of RXRA-null mice which die in utero (vide infra), suggesting a degree of functional redundancy [25].

The DNA-binding sites for RARs and RXRs are known as retinoic acid response elements (RAREs) and contain direct repeats (DR) of 5'-AGGTCA-3' separated by one to five base pairs (termed DR1-DR5) [48, 49]. DRs (DR1-5) determine RA-activated RAR-RXR complex target gene expression. For example, DR5-containing genes display transcriptional activation, whereas DR1-containing genes display transcriptional repression [50]. So far, a wide variety of the RAR- and RXR-regulated genes have been shown to influence many cellular processes—e.g., the cellular uptake of RA (*Crbp1/2* and *Crabp1/2*), RA catabolism (*Cyp26a1*), RA nuclear receptor beta (Rarb), mammalian embryonic pattern formation through the homeobox (Hox) family (*Hoxa1*, *Hoxb1*, *Hoxb4*, and *Hoxd4*), and organ growth/development (*Pitx2*, *Drd2*, *Gad67*, *Fgf8*, and *Pdx1*) [51–53].

### The retinoic acid pathway regulates eye development

RA-signaling in mammalian eye development has been previously reviewed [54]. As such, we will focus on ocular developmental diseases associated with perturbed RA-signaling.

The process of eye development is largely conserved among chordates—including zebrafish, mice, and humans [55, 56]. Mouse eye development begins at E8.0, at which time the optic vesicle forms on the cephalic neural folds [57]. The optic vesicle then begins to migrate towards the surface ectoderm until, at E9.0, the two ectodermal layers come into contact and begin to thicken. This contact initiates activation of a cascade of transcription factors (e.g., SIX3 and PAX6) [*reviewed in* [58]] and signaling pathways [e.g., BMP and RA (*reviewed in* [59]].

The optic vesicle then invaginates into the optic cup, and the surface ectoderm subsequently invaginates to form the lens placode. As the lens placode continues to invaginate, asymmetric cell growth then leads to formation of the lens pit, with the ultimate formation of the lens vesicle by E11. Epithelial cells located at the anterior portion of the lens vesicle maintain their epithelial identity and proliferative nature, whereas epithelial cells at the posterior lens vesicle differentiate into fiber cells and ultimately become primary lens fiber cells. The inner layer of the optic vesicle then becomes the neuroretina, while the outer layer becomes the RPE [60].

From mouse neuroretina, several neuronal subtypes (i.e., retinal ganglion cells, amacrine cells, horizontal cells, bipolar cells, photoreceptor cells) and Müller cells begin forming at E11. Corneal development begins when the lens stalk connecting the lens vesicle to the surface ectoderm is severed. The resulting space is rapidly filled by invading cells from perinuclear mesenchyme. Mesenchymal cells nearest the lens vesicle then form the corneal endothelium, while cells farthest from the lens vesicle form the corneal epithelium. Cells located between these two layers form the corneal stroma from which corneal keratocytes differentiate.

Corneal development is maintained by a constant influx of cells from the periocular mesenchyme (POM). Lens formation continues, with secondary fiber differentiation in the mouse beginning at the lens equator at E13.5-E14.5 (secondary fiber cell differentiation occurs throughout adulthood). Anterior segment development is completed by the anterior edge of the optic cup (which forms the epithelium of the iris and ciliary body), and migrating cells from the POM form stroma of the iris and ciliary body. Lastly, the trabecular meshwork is formed from migrating mesenchymal cells. Eye morphogenesis is largely conserved in mouse, zebrafish, and humans, but the process in zebrafish occurs in a much shorter time frame [56, 61].

Studies in animal models have revealed a requirement for RA-signaling in normal eye development. Beginning in the mid-twentieth century, research highlighted the importance of dietary vitamin A in maintaining rodent eye development [3, 62]. Rats born to mothers maintained on a vitamin A-deficient diet displayed a multitude of ocular defects—including retina infolding, coloboma, microphthalmia, and anophthalmia (a syndrome ultimately termed vitamin A deficiency)—that could be rescued by vitamin A supplementation during embryonic development [3, 62]. However, the time of supplementation was critical, in that supplementation before E13.0 could rescue the ocular phenotypes whereas supplementation after E13.0 could not completely rescue eye development [3]. These studies demonstrated that vitamin A (and ultimately RA-signaling) is required for specific events in eye development, i.e., optic cup formation, anterior segment formation.

As noted, RA-signaling is modulated by several enzymes and dependent on RARs and RXRs (Fig. 1). Therefore, an alteration in any one of these proteins may perturb RA-signaling and affect normal eye development. Animal models used to investigate the RA-signaling pathway will be discussed in the order that the proteins appear within the pathway—starting with RBP4 and finishing with RARs and RXRs (Fig. 1).

In zebrafish, decreased stra6 (e.g., by morpholino knockdown) causes reduced eye size, despite the formation of all retinal layers [63]. In mouse, RDH10 was identified as essential for normal eye development [24]. RDH10-deficient mice lack the cornea and ventral half of the retina and exhibit hypoplastic lenses. RDH10-depleted zebrafish display a mild RA loss of function phenotype [64], perhaps due to their ability to produce retinaldehyde through bcox [29]. A morpholino-mediated inhibition of *bcox* results in microphthalmic zebrafish with a diminished size of the ventral prospective retina tissue [29].

**Fig. 2:**
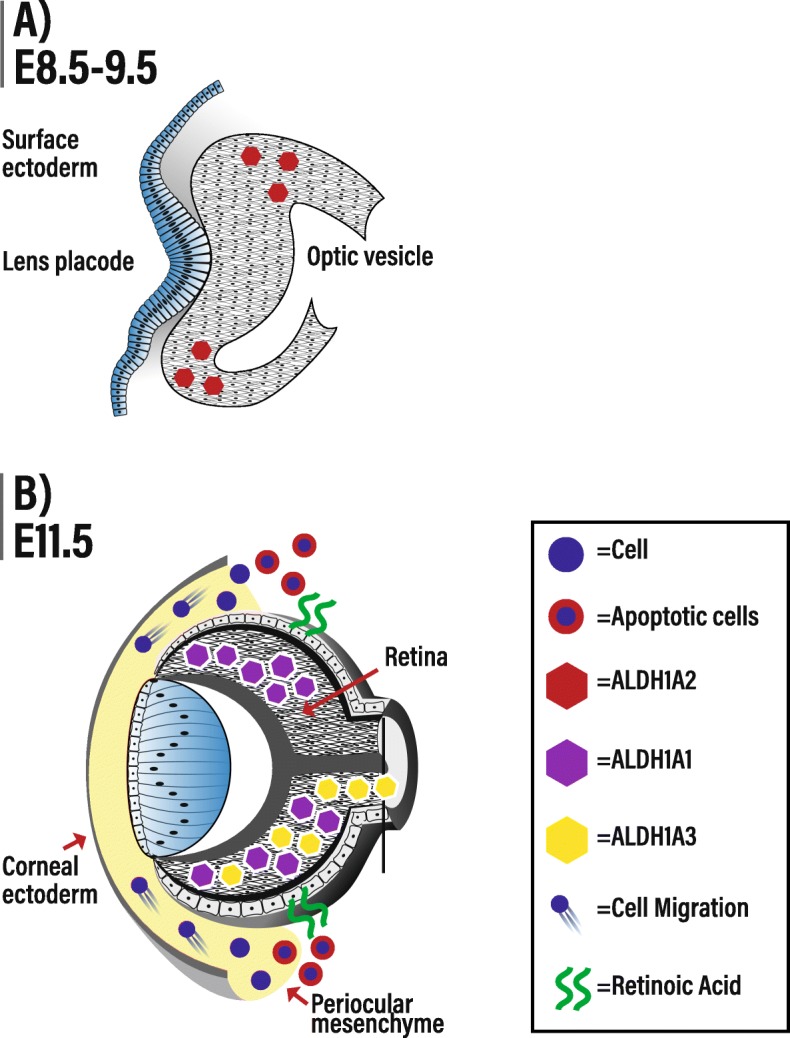
The retinoic acid (RA)-signaling pathway and eye development. Key events in mouse eye development that are regulated by RA-signaling are shown. **a** Between E8.5 and E9.5, ALDH1A2 is expressed in the optic vesicle and is required for optic cup formation [65]. **b** Starting at E10.5, RA produced by ALDH1A1 and ALDH1A3 within the developing retina diffuses (shown by green arrows) into periocular mesenchyme (POM) where it activates RARBs/RARGs on POM cells to induce cell apoptosis and thus control the migration of POM cells to the anterior eye. This diagram represents conditions at E11.5 [inspired by [27]. Abbreviations: ALDH1A1, aldehyde dehydrogenase 1 family member A1; ALDH1A2, aldehyde dehydrogenase 1 family member A2; ALDH1A3, aldehyde dehydrogenase 1 family member A3; E, embryonic day

In mice, ALDHs are differentially required at various stages of eye development. ALDH1A2 is only expressed in the murine eye between E8.5 and E9.5 and is required for optic cup formation (Fig. 2a) [65]. In contrast, ALDH1A1 and ALDH1A3 are respectively expressed in dorsal and ventral retina from E10.5 onwards [27] (Fig. 2b). *Aldh1a1*-null mice exhibit no developmental ocular phenotype, likely due to compensation in RA-signaling by ALDH1A3 [27, 28]. *Aldh1a3-*null mice display developmental ocular phenotypes—resulting as ventral rotation of the lens, persistence of the primary vitreous, and thickening of the ventral POM [27, 66]. *Aldh1a1/Aldh1a3*-null mice display all of the same phenotypes as *Aldh1a3*-null mice, but with greater severity; this would suggest that some of the loss of RA-signaling induced by the genetic ablation of *Aldh1a3* is compensated by *Aldh1a1* [27]. RA influences mammalian ocular development in a paracrine fashion; RA produced in the retina by ALDH1A1 and ALDH1A3 is secreted and acts on cells in the POM where it regulates expression of genes important for apoptosis and corneal morphogenesis and cell specification—*Eya2* and *Pitx2*, respectively (Fig. 2b) [27, 67, 68].

Disruption in RA-signaling permits overgrowth of POM cells, which adversely affects normal anterior segment development [27, 28]. Ectopic lens expression of CRABP1 results in lenses with impaired secondary fiber cell differentiation (i.e., failure to lose nuclei) and a flattening of the anterior side of the fiber cells [69]. ATRA binds to and activates the RXR/RAR complex, which enables activation or repression of RARES (Fig. 1). Compound *Rar* gene deletions (for example, the deletion of both *Rara* and *Rarb*) result in aberrant ocular developmental phenotypes—including microphthalmia, coloboma, lens abnormalities, and retinal dysplasia and degeneration [70, 71].

Compound *Rxra, Rxra/Rarg*, and *Rxra/Rara* null mice all show ocular developmental abnormalities—including ventral rotation of the lens, thicker corneas, shorter ventral retinae, and coloboma [72]. Involvement of RA-signaling in maintenance of POM cellular proliferation was confirmed by the conditional deletion of *Rara*, *Rarb*, and *Rarg* in neural crest cells; these knockout animals have impaired ocular development phenotypes similar to the previously described ALDH-deficient mice [68]. Collectively, these animal models have provided compelling evidence in support of an important role for RA-signaling in ocular development.

Mutations in genes involved in RA-signaling in humans are associated with developmental diseases—including the ocular developmental diseases, such as microphthalmia, anophthalmia, and coloboma (collectively called MAC disease), and Mathew-Wood Syndrome (Fig. 1). Linkage analysis and whole-exome sequencing have identified mutations in *RBP4* in patients with MAC disease [73, 74]. Dominant-negative mutations in *RBP4* increase RBP4 affinity for STRA6; this nonproductive occupation of STRA6 hinders delivery of vitamin A to the fetus. Maternal inheritance of *RBP4* mutations and a lack of maternal dietary retinoids predispose the fetus to MAC disease [73]. Mutations in *STRA6* are associated with both syndromal (Mathew-Wood Syndrome) and non-syndromal MAC disease [75–78]. A double-nucleotide polymorphism that causes a nonsynonymous change from glycine to lysine in a highly conserved region of the STRA6 protein was identified in MAC patients; this mutation almost completely abolishes cellular uptake of vitamin A [75].

Homozygous nonsense mutations, missense, and splice-site mutations in *ALDH1A3* are associated with microphthalmia [79–81]. Co-transfection of wild-type and mutated human *ALDH1A3* (c.265C>T and c.1477G>C) revealed that the mutated ALDH1A3 protein is likely unstable and subject to proteasomal degradation [79]. Mutations in *RARB* have been identified in patients with syndromic MAC [i.e., pulmonary hypoplasia/agenesis, diaphragmatic hernia/eventration, anophthalmia/microphthalmia, and cardiac defect (PDAC)] [82, 83]. Evaluation of these mutations have indicated that both gain-of-function and dominant-negative mutations within *RARB* can cause PDAC syndrome [82]. A recent report identified a de novo mutation in *RARA* in a coloboma patient which is hypothesized to impair the interaction between RA and RARA [84].

It is clear that RA-signaling is similarly important for zebrafish, mouse, and human eye development. Given that it is unethical to investigate the role of RA-signaling in human eye development in a manner similar to that in animal models, it is currently unknown exactly how RA-signaling might impact human eye development. However, the conserved nature of RA-signaling and eye development across chordates suggests that RA-signaling is also very likely to act in a paracrine fashion to regulate eye development in humans.

### Deleterious clinical variations in the RA pathway

The ExAC database (comprising whole-exome sequencing of more than 60,000 individuals) was published in 2016 [85]; it was recently expanded to include ~ 140,000 individuals [16]. Using this resource, it is now possible to gain insights into the necessity of various components of the RA pathway in humans and to explore the existence of nodes in the pathway that are of potential importance.

A gene with a high pLI score [85] would suggest that individuals who inherit loss-of-function (LOF) mutations in that gene will inherit a survival disadvantage. Analysis of pLI scores of genes involved in the RA-signaling pathway (Table 1) reveals that certain genes in this pathway are crucial for human embryogenesis and life—data that are consistent with mouse studies (Table 2). The RA-signaling pathway is essential for life, with 30% of RA-signaling pathway genes being categorized as LOF-intolerant. This is in striking contrast to 17% of all known genes being categorized as LOF-intolerant [85]. Several of the LOF-intolerant RA-signaling pathway genes have no known associated human disease, i.e., *RDH10, RXRA, RXRB* (Table 1); this would be predicted given the severe embryonic lethality observed in transgenic mouse models in which these three genes have been knocked out (Table 2).

**Table 1:** Genes involved in the retinoic acid synthesis, degradation, and signaling in humans

Gene	Diseases linked to mutations in gene	OMIM reference number	Probability loss-of-function (pLOF) allele counts	Number of individuals homozygote for a pLOF allele	pLI
*ADH1*	Fetal alcohol syndrome (ADH1A) [86]; increased alcohol sensitivity (ADH1B) [87]; ethanol-induced; cutaneous erythema [88]	103700 and 103720 and 103730	131	1	0
*ADH7*		600086	106	1	0
*ALDH1A1*	Fatty liver [89], alcohol flushing [90]	100640	6	0	0.95
*ALDH1A2*		603687	20	0	0.36
*ALDH1A3*	Bilateral severe microphthalmia, anophthalmia [79] [80]	600463	53	0	0.14
*ALDH1B1*		100670	> 3000	71	0
*ALDH8A1*		606467	49	0	0
*CYP1B1*	Primary congenital glaucoma [91, 92]; Juvenile/Adult POAG [93]; Peters Anomaly [94]	601771	235	0	0
*CYP26A1*	Decreased metabolism of ATRA (cell culture) [95]	602239	45	0	0
*CYP26B1*	Radiohumeral fusions and other skeletal and craniofacial anomalies [96]	605207	90	0	0.98
*CYP26C1*	Focal facial dermal dysplasia [97]	608428	663	1	0
*CRABP1*	Upregulation of CRABP1 contributes to retinoid resistance in leukemia [98]	180230	10	0	0.01
*CRABP2*		180231	73	0	0.00
*FABP5*	Increased in psoriatic skin lesions [99]	605168	13	0	0.05
*RARA*	~ Acute promyelocytic leukemia (APL) during gain of function/translocation [100]	180240	75	2	0.96
*RARB*	Premalignant oral lesions, microphthalmia, diaphragmatic hernia, pulmonary hypoplasia, and cardiac abnormalities [76, 82]	180220	11	0	1.00
*RARG*	~ APL during gain of function/translocation [101]	180190	48	0	0.99
*RBP4*	Micropthalmia, anophthalmia, coloboma (MAC) [73, 74], night blindness and retinal dystrophy [102]	180250	8	0	0.52
*RDH5*	Fundus albipunctatus [103, 104]	601617	109	0	0
*RDH10*		607599	2	0	0.99
*RXRA*		180245	0	0	1.00
*RXRB*		180246	20	0	1.00
*RXRG*		180247	6	0	0.42
*STRA6*	Syndromal and non-syndromal MAC [75–78]	610745	265	4	0

**Table 2:** Mouse phenotypes for genes involved in the retinoic acid synthesis, degradation, and signaling

Gene	Synonyms	MGI identification number	Mouse knockout phenotypes (homozygous null mutations)
*Adh1*		87921	Impaired metabolism of (and sensitivity to) ethanol and retinol [105–107]
*Adh7*	ADH4	87926	Defective ethanol clearance and reduced metabolism of retinal to RA [105]
*Aldh1a1*	RALDH1	1353450	Increased energy dissipation, insulin resistance, diet-induced obesity resistance [108], significantly reduced ability to convert retinol to retinoic acid in the liver [109]
*Aldh1a2*	RALDH2	107928	Devoid of retinoic acid, die by E10.5 with impaired hindbrain development, failure to turn, lack of limb buds, heart abnormalities, reduced otocysts and a truncated frontonasal region [110–114]
*Aldh1a3*	RALDH3	1861722	Neonatal death [115], persistent hyperplastic primary vitreous, thick neural retina and no vitreum [28], choanal atresia, ethmoturbinal hypoplasia, ventral lens rotation, short ventral retina, and no Harderian gland [66]
*Aldh1b1*	ALDHX	1919785	Increased fasting circulating glucose levels and decreased blood acetaldehyde clearance [116], defects in beta cell development and functionality, glucose intolerance, age-dependent hyperglycemia, and insulin resistance [117]
*Aldh8a1*	ALDH12 or RALDH4	2653900	None Found
*Cyp1b1*		88590	Protected from the acute bone marrow cytotoxic and preleukemic effects of DMBA [118], show a decreased incidence of DMBA-induced lymphomas [119], ocular drainage structure abnormalities (~ POAG) [120]
*Cyp26a1*		1096359	Mid-late gestation lethal and exhibit spina bifida, caudal agenesis, and abnormalities of the kidneys, urogenital tract, hindgut, cervical vertebrae, and rostral hindbrain [121, 122], rescued by partial *Aldh1a2* deletion [42]
*Cyp26b1*		2176159	Lethal immediately after birth exhibiting respiratory distress [123], limb morphogenesis and proximal-distal patterning is disrupted in homozygous null fetuses [123, 124], abnormal spermatogenesis/oogenesis [125], arrested hair follicle development [126]
*Cyp26c1*		2679699	Viable, exhibit normal CNS development with no apparent anatomical defects [127]
*Crabp1*	CRABP-I	88490	Phenotypically normal and fertile [128]
*Crabp2*	CRABP-II		Postaxial polydactyly [129]
*Fabp5*		101790	Impaired skin barrier function [130], resistance to diet-induced obesity (decreased adipose tissue and improved glucose tolerance) [131], and impaired cognitive function [132, 133]
*Rara*	NR1B1	97856	High neonatal mortality due to maternal cannibalization, failure to thrive, and excess mortality during the postnatal period, male survivors exhibit testicular degeneration [134–136]
*Rarb*	NR1B2	97857	Reduced growth [137], locomotion abnormalities [138]
*Rarg*	NR1B3	97858	Stunted growth, homeotic transformations of the rostral axial skeleton and tracheal cartilage, Harderian gland agenesis, high postnatal mortality, and male sterility [135, 139], splenomegaly, and myeloproliferative disease, abnormal granulocytes [140]
*Rbp1*	CRBPI	97876	Increased adiposity, increased PPAR-gamma target gene expression [141], increased Rpb2 and Crabp2, elevated pancreatic RA [142], increased susceptibility to a diet deficient in vitamin A [143]
*Rbp2*	CRBPII	97877	Pups of homozygous dams fed a marginal retinol diet show increased neonatal lethality due to inadequate retinal transport to the fetus, abnormal retinol level and vitamin absorption [144]
*Rbp4*		97879	Impaired retinal function in first month of life [145], insulin sensitivity [146]
*Rdh5*		1201412	Impaired dark adaptation and at high bleaching levels, large increase in 11-cis-retinyl ester concentration [147]
*Rdh10*		1924238	Mid-gestational lethality, reduced RA-signaling and abnormal limb, craniofacial, somite and cardiac morphology including microphthalmia [24] and dorsal pancreas agenesis [148]
*Rxra*	NR2B1	98214	Multiple organ defects and die of cardiac failure by E14.5, eye defects (retinal abnormalities, late corneal opacity), placental defects [149–153] .
*Rxrb*	NR2B2	98215	Partial embryonic and perinatal lethality, and surviving adult males are sterile due to defects in spermatogenesis [154]
*Rxrg*	NR2B3	98216	Neuron reduction in striatum, premature death and altered responses to the administration of dopamine antagonists [155]
*Stra6*		107742	Seven-fold reduction in total ocular retinoids, photoreceptor anomalies, abnormal RPE, sclera, and choroid [20]

A total of eight RA-signaling pathway genes [85] have markedly high pLI scores (pLI > 0.9), i.e., *ALDH1A1*, *CYP26B1*, *RARA*, *RARB*, *RARG*, *RXRA*, *RXRB*, and *RDH10* (Table 1)*.* These genes are therefore essential for life, e.g., DNA-binding functions and crucial for morphogenesis [156, 157].

pLI predictions and animal models are in agreement for one gene in particular—*ALDH1A2.* Humans are LOF-tolerant (pLI = 0.36) (Table 1) and *Aldh1a2* heterozygous null mice are viable. This highlights an important feature of pLI scores; they *predict* the probability of haploinsufficiency intolerance [158]. However, it is important to note that *Aldh1a2*-null mice experience embryonic lethality by E10.5 (Table 2) [111].

Despite the high level of conservation in the RA-signaling pathway between humans and mice, discrepancies in the extent of indispensability of RA-signaling pathway genes exist. *ALDH1A1* is intolerant of LOF mutations (pLI = 0.95) in humans (Table 1) whereas *Aldh1a1* is dispensible in mice [27]. This may be related to loss of *Aldh1a1* being compensated in mice by *Aldh1a7*—which is an *Aldh1a1* gene duplication found in rodents but not in humans [159].

In mice, *Cyp26b1* deletion is lethal immediately after birth [123, 124], whereas humans with *CYP26B1* mutations can live to adulthood [96]. *Cyp26a1*-null mice experience embryonic lethality (Table 2), while human CYP26A1 is LOF-tolerant (pLI = 0) (Table 1). Differences between mouse [160] and human [161] *CYP26A1* tissue expression may explain the differential requirements for life. For example, human MAP2-positive neurons in the human dentate gyrus express CYP26A1, whereas rat and mouse MAP2-positive neurons do not [162]. Further, human MAP2-positive neurons express ALDH1A2 along with CYP26A1, suggesting that RA acts in an autocrine fashion in these cells, as opposed to the paracrine fashion found in rodents. This may explain the differences in the requirement for life. Differences between mouse and human underscores the need for caution when generalizing the requirement for life of RA-signaling pathway genes across chordates. Despite this, mice still hold great utility as an experimental model when investigating the minutiae of the RA-signaling pathway.

Differences between animal models and humans can be further explained by incomplete penetrance in human—possibly due to differences in mutation type, variations in gene expression, epigenetic changes, age, sex, or copy number variations [163]. Often, experimental models used for investigation of the RA-signaling pathway rely on transgenic mice in which a pathway gene is completely ablated. This situation may not be representative of humans in whom gene mutations commonly result in lowered gene activity rather than zero expression. In addition, important differences during development in the patterns of expression of many genes and their pathways exist between mouse and human [164–166]. As was highlighted above, differences in the timing and/or location of RA-signaling pathway genes result in vast differences in this critical pathway—e.g., autocrine vs. paracrine signaling, embryonic or postnatal lethality, and tissue-specific expression (or lack thereof). All of these can contribute to the discrepancies between mouse models for the RA-signaling pathway genes, observed human diseases, and the pLI scores.

### Future studies of the retinoic acid signaling pathway

By means of analysis of genetic intolerance, we can pinpoint certain members of the RA-signaling pathway that are likely to be essential for human life. Clearly, it is important that these members need to be better understood. This can be achieved by generating hypomorphic mutations in mice, i.e., protein function is diminished rather than ablated. Hypomorphic mutations in mice can be studied via the introduction of single-nucleotide variants (SNVs) using CRISPR/*Cas9* technology [*reviewed in* [167]]. This approach was recently used to study hypomorphic mutations in a humanized CYP3A5*1 mouse model [168]. These humanized mice with hypomorphic mutations are likely to be better models of human diseases associated with altered RA-signaling pathways.

While CRISPR/*Cas9* can be effectively used for modeling human diseases in mice, such studies can be prohibitively inefficient in that they use advanced techniques, need specialized equipment, and require at least 3 months to generate knockin/knockout mice [169]. Zebrafish represent an alternative in which high-throughput screening can be used to investigate mutations in genes of the RA-signaling pathway that have been identified in humans [[170] and *reviewed in* [171]]. Zebrafish are more efficient than mice for such CRISPR/*Cas9* experimental approaches for several reasons: they have a shorter generation time, produce more offspring, and are less expensive to maintain [172–175]. CRISPR/*Cas9* was recently used in zebrafish to generate a humanized model of renal agenesis in which GREB1 like retinoic acid receptor coactivator (GREB1L) was identified as a coactivator of RAR genes [176]. It is expected that similar approaches will be used in future studies to manipulate the RA-signaling pathway and thereby enhance our understanding of RA-signaling in human physiology and pathophysiology.

## Conclusions

For centuries, the importance of dietary vitamin A to human health has been known. Ancient civilizations unknowingly used homeopathic remedies in which vitamin A was the main active ingredient. It was not until the turn of the twentieth century that our more nuanced understanding of the role for vitamin A in human health began to take shape. Pioneering animal studies determined that vitamin A was critical for embryogenesis, eye development, and identified retinoids as derivatives of vitamin A. Decades later, our understanding of the RA-signaling pathway has grown significantly and now includes a more comprehensive knowledge of retinol cellular uptake and oxidation, RA catabolism, nuclear receptor (RAR/RXR) activation, and nuclear receptor gene targets—and the importance of the RA-signaling pathway for eye development. By leveraging information gained from large-scale human whole-exome sequencing efforts (e.g., ExAC and gnomAD), our understanding about the importance of the RA-signaling pathway to human health is improving. This was underscored by the high number of genes within this pathway with large pLI scores. While transgenic mouse models have provided valuable insights into the details of the RA pathway, discrepancies between the human and mouse data underscore the need for care when generalizing results from animal studies to humans.

Animal models will continue to enhance our understanding of the RA-signaling pathway under physiological and pathophysiological conditions. Exciting new models and techniques (e.g., Zebrafish, CRISPR/*Cas9*, hypomorphic mutations) will allow a more nuanced examination of this pathway. These will allow the pathophysiological consequences of individual human mutations in the RA-signaling pathway to be explored. In summary, the RA-signaling pathway play a critical role in embryogenesis, eye development, and are required for life. We should anticipate fascinating new insights into this pathway in the coming years.

## Data Availability

All data analyzed in this review are included in this published article: Karczewski KJ, Francioli LC, Tiao G, Cummings BB, Alföldi J, Wang Q, et al. Variation across 141,456 human exomes and genomes reveals the spectrum of loss-of-function intolerance across human protein-coding genes. bioRxiv. 2019:531210; doi: 10.1101/531210

## Abbreviations

ADH1: Alcohol dehydrogenase 1
ADH7: Alcohol dehydrogenase 7
ALDH1A1: Aldehyde dehydrogenase 1 family member A1
ALDH1A2: Aldehyde dehydrogenase 1 family member A2
ALDH1A3: Aldehyde dehydrogenase 1 family member A3
ATRA: All-*trans*-retinoic acid
CRBP: Cellular retinol-binding protein
CYP26A1: Cytochrome P450 family 26 subfamily A member 1
CYP26B1: Cytochrome P450 family 26 subfamily B member 1
CYP26C1: Cytochrome P450 family 26 subfamily C member 1
DRs: Direct repeats
ExAC: Exome Aggregation Consortium
gnomAD: The Genome Aggregation Database
GREB1L: GREB1 like retinoic acid receptor coactivator
LOF: Loss of function
MAC: Microphthalmia, anophthalmia, and coloboma
pLI: Probability loss of function intolerance
POM: Periocular mesenchyme
PTVs: Protein-truncated variants
RA: Retinoic acid
RAR: Retinoic acid receptor
RARE: Retinoic acid response elements
RBP4: Retinol-binding protein 4
RDH10: Retinol dehydrogenase 10
RDH5: Retinol dehydrogenase 5
RPE: Retinal pigment epithelium
RXR: Retinoid X receptor
STRA6: Stimulated by retinoic acid 6
WES: Whole-exome sequencing
